# Could Nitazoxanide Be Added to Other Essential Medicines for Integrated Neglected Tropical Disease Control and Elimination?

**DOI:** 10.1371/journal.pntd.0002758

**Published:** 2014-03-27

**Authors:** Peter J. Hotez

**Affiliations:** 1 Sabin Vaccine Institute and Texas Children's Hospital Center for Vaccine Development, Departments of Pediatrics and Molecular Virology and Microbiology, National School of Tropical Medicine, Baylor College of Medicine, Houston, Texas, United States of America; 2 James A. Baker III Institute for Public Policy, Rice University, Houston, Texas, United States of America

*New findings from two Gates Foundation–supported studies—the Global Enteric Multicenter Study and the Global Burden of Disease Study 2010—suggest the possible importance of adding coverage for intestinal protozoan infections as part of World Health Organization preventive chemotherapy initiatives.*

In 2011, the World Health Organization (WHO) determined that more than 700 million people were treated with at least one essential medicine for neglected tropical diseases (NTDs) under the auspices of a global preventive chemotherapy initiative [1], [2]. However, a total of at least 1.9 billion people require annual preventive chemotherapy [1], [2], so these efforts will need to be greatly expanded in order to meet NTD control and elimination targets as outlined in the 2012 London Declaration and the 2013 World Health Assembly resolution for these diseases [3].

The original “rapid-impact” package of NTD interventions targeted up to seven NTDs highly endemic to sub-Saharan Africa, including the three soil-transmitted helminthiases, schistosomiasis, lymphatic filariasis, onchocerciasis, and trachoma, and was comprised of up to four essential NTD medicines that could include a benzimidazole anthelminthic drug (i.e., mebendazole or albendazole), ivermectin, praziquantel, and/or azithromycin [4], [5]. However, it was quickly noted that either the entire rapid-impact package or some component thereof had applicability outside of Africa (with modifications depending on the specific NTDs being targeted) [6]. By controlling or eliminating the seven major NTDs, this approach could potentially effect a global disease burden reduction almost as important as HIV/AIDS, tuberculosis, or malaria control [7], [8].

As global preventive chemotherapy efforts expanded, it also became apparent that they could produce important collateral public health benefits that were not originally anticipated, including overall reductions in child mortality from the azithromycin component [9] and coverage for additional NTDs such as food-borne trematodiases, scabies, and yaws [3], [10], [11]. There are equally important efforts underway to broaden the interventions to include water, sanitation, and hygiene (WASH) initiatives [12]. Thus, in the decade since rapid impact was originally proposed, there are new uses and approaches for preventive chemotherapy.

In the last year, two important studies were published that could alter how we think about current preventive chemotherapy approaches. The first, known as the Global Enteric Multicenter Study (GEMS) for diarrheal diseases, made the surprising finding that cryptosporidiosis is one of the most important causes of infectious diarrhea in children in developing countries [13]. The second is the Global Burden of Disease Study 2010 (GBD 2010), which found that, together, cryptosporidiosis and amoebiasis exceed the disease burden–as measured in disability-adjusted life years (DALYs) or in deaths—of any helminth infection now currently being targeted for preventive chemotherapy (Table 1) [14], [15]. Although there are important disagreements in the NTD community about whether the DALYs for helminth infections (and other NTDs) were underestimated [16], both GEMS and GBD 2010 provide important information to our community that we need to consider in deciding whether it is possible to add coverage for cryptosporidiosis and amoebiasis as part of global preventive chemotherapy efforts.

**Table 1:** Ranking of leading parasitic diseases by DALYs and deaths (modified from refs [14] and [15]).

Disease	Type of Parasitic Disease	DALYs	Deaths
Cryptosporidiosis + Amoebiasis	Protozoan	10.5 million	155,300
Cryptosporidiosis	Protozoan	8.4 million	99,800
Soil-transmitted helminth infections: Hookworm, Ascariasis, and Trichuriasis	Helminth	5.2 million	2,700
Schistosomiasis	Helminth	3.3 million	11,700
Leishmaniasis	Protozoan	3.3 million	51,600
Hookworm infection	Helminth	3.2 million	-
Lymphatic filariasis	Helminth	2.8 million	-
Amoebiasis	Protozoan	2.2 million	2,700
Food-borne Trematodiases	Helminth	1.9 million	-

**Figure 1:**
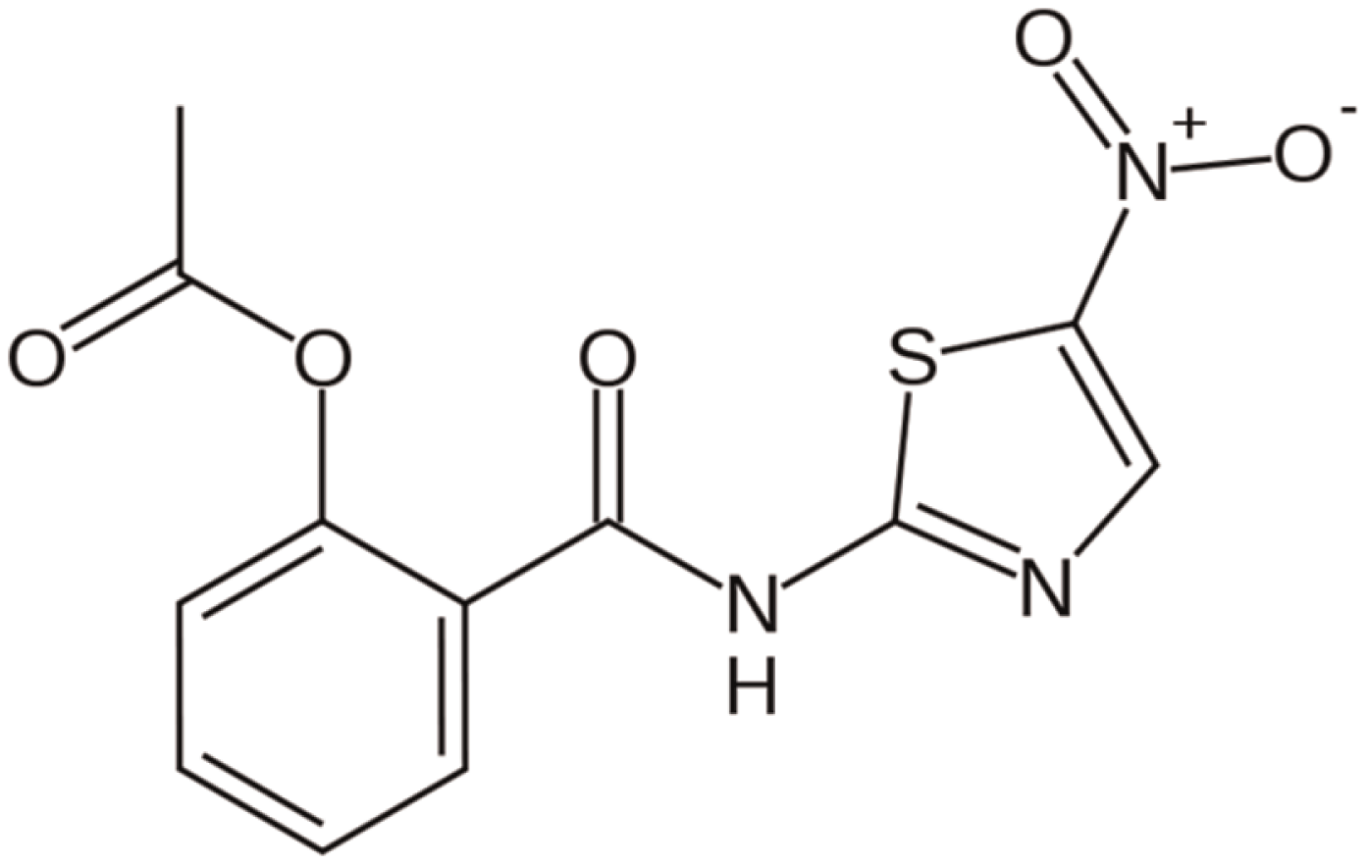
Chemical structure of nitazoxanide. Image source: http://commons.wikimedia.org/wiki/File:Nitazoxanide.svg, accessed December 26, 2013.

A potential candidate drug for use in mass drug administration programs to target intestinal protozoa is the nitrothiazole benzamide drug, nitazoxanide (Figure 1) [17]. The development program for nitazoxanide was led by Jean Francois Rossignol in the 1970s, initially as a veterinary anthelminthic agent, but the drug was subsequently shown to be active against intestinal protozoa and some human helminths, as well as anaerobic bacteria [17]. It was approved in 2002 by the United States Food and Drug Administration, initially as an oral suspension for pediatric use (100 mg/5 ml) against cryptosporidiosis and giardiasis, and subsequently as 500 mg tablets for adults [17]. According to The Medical Letter, the recommended therapeutic dosage is administered over three days [18].

Although the initial indication for nitazoxanide was for cryptosporidiosis and giardiasis, subsequent investigative research has revealed that the drug is effective for amoebiasis caused by *Entamoeba histolytica* and could be used to treat both invasive intestinal amoebiasis and colonization with *E. histolytica* [19]. Moreover, nitazoxanide is active against a number of nonprotozoan parasites, including the intestinal tapeworm *Hymenolepis nana* [20], with variable efficacy against the soil-transmitted nematodes [21]–[23]. It was recently shown to be an ineffective drug for the treatment of human trichuriasis [24]. Nitazoxanide has also shown promise for the treatment of diarrhea caused by the bacterium *Clostridium dificile* [25], [26] and for viral gastroenteritis caused by rotavirus, norovirus, and possibly other enteric viruses [27]–[30]. The drug or its derivatives has shown further promise as an innovative treatment for hepatitis C infection [31].

Because of its broad-spectrum activity against a variety of intestinal pathogens, there has been interest in evaluating nitazoxanide as a potential agent for public health control. Among Mexican children, the drug showed promise for reducing the burden of a variety of intestinal parasites [21], while Rossignol et al. have proposed nitazoxanide for the empiric treatment of pediatric diarrhea [32]. To date, the medicine has an excellent overall safety spectrum, with occasional gastrointestinal disturbances and headache, and rarely (according to The Medical Letter), allergies, yellow discoloration of the sclera, and other rare side effects [18].

The possibility of adding nitazoxanide to current regimens used for WHO preventive chemotherapy initiatives as a means to broaden coverage against intestinal protozoa and possibly other pathogens has the potential of significantly increasing preventive chemotherapy's impact on reducing global disease burdens of parasitic infections and NTDs. However, to even consider adding nitazoxanide, there are quite a few operational research questions that would need to be addressed. Among them is that most likely nitazoxanide would need to be administered alongside other NTD drugs in the rapid-impact package as an annual single dose. It is unclear whether a single dose of 500 mg of the drug, or even a 1 g dose as used in a trichuriasis field study in Pemba, Tanzania [24], would have a significant impact in terms of reducing intestinal parasitism associated with *Cryptosporidium partum* or *E. histolytica*, especially in field conditions in a highly disease-endemic country of Africa or Asia.

Another important issue is selecting the optimal targeted age group. According to the GEMS study, cryptosporidiosis has its most important impact on preschool children, especially children under the age of two [13]. Today, a significant percentage of deworming (i.e., mass drug administration of a benzimidazole anthelminthic agent for soil-transmitted helminth infections) is conducted on preschool-aged children—more than 250 million preschool children were treated in 2011 [33], and there is a rationale for extending schistosomiasis coverage with praziquantel for preschool-aged children [34]. Is there an opportunity to simultaneously administer nitazoxanide with these anthelminthics? An alternative possibility is the co-administration of nitazoxanide with intermittent preventive treatment of malaria in infants (IPTi) or children (IPTc) [35].

Added concerns are the drug compatibilities and, of course, the safety and efficacy of co-administering NTD medicines with nitazoxanide. There is also the issue of cost—could nitazoxanide join other essential NTD medicines as a donated drug? Certainly the impact of providing nitazoxanide in programs of mass drug administration must also be further evaluated for the potential to promote drug resistance.

Such aspects would have to be carefully addressed in well-conducted and supervised programs of operational research in appropriately resource-poor settings. However, the potential benefits of adding cryptosporidiosis and amoebiasis targets to current preventive chemotherapy regimens may be sufficiently important to warrant next steps in evaluating nitazoxanide in this context.
